# Electroacupuncture for patients with spasticity after stroke: A protocol for systematic review and meta-analysis

**DOI:** 10.1097/MD.0000000000024859

**Published:** 2021-02-19

**Authors:** Kang Yang, Hongshi Zhang, Guanyu Hu, Ye Zhang, Huijuan Lou, Meng Meng, Yufeng Wang, Deyu Cong

**Affiliations:** a Department of Acupuncture and Tuina, Changchun University of Chinese Medicine; b Department of Tuina, Traditional Chinese Medicine Hospital of Jilin Province, China.

**Keywords:** electroacupuncture, protocol, spasticity after stroke, systematic review

## Abstract

**Background:**

The purpose of this paper is to evaluate the effectiveness and safety of electroacupuncture in the treatment of spasticity after stroke.

**Methods:**

We will electronically search PubMed, Medline, Embase, Web of Science, the Cochrane Central Register of Controlled Trials, China National Knowledge Infrastructure, Chinese Biomedical Literature Database, Chinese Scientific Journal Database, and Wan-Fang Database from the date of creation to November 2020. In addition, we will manually retrieve other resources including the reference lists of identified publications, conference articles, and gray literature. The clinical randomized controlled trials or quasi-randomized controlled trials related to electroacupuncture in the treatment of spasticity after stroke will be included in the study. The language is limited to Chinese and English. Research selection, data extraction, and research quality assessment will be independently completed by 2 researchers. Data were synthesized by using a fixed effect model or random effect model depend on the heterogeneity test. The modified Ashworth scale was the primary outcomes. Simplified Fugl–Meyer assessment scale (FMA), Stroke specific quality of life scale (SS-QOL) and adverse events will also be assessed as secondary outcomes. RevMan V.5.3 statistical software will be used for meta-analysis. If it is not appropriate for a meta- analysis, then a descriptive analysis will be conducted. Data synthesis will use the risk ratio and the standardized or weighted average difference of continuous data to represent the results.

**Results:**

This study will provide a high-quality synthesis to assess the effectiveness and safety of electroacupuncture in the treatment of spasticity after stroke.

**Conclusion:**

This systematic review will provide evidence to judge whether electroacupuncture is an effective and safety intervention for patients with spasticity after stroke.

**Ethics and dissemination:**

The protocol of the systematic review does not require ethical approval because it does not involve humans. We will publish this article in peer-reviewed journals and presented at relevant conferences.

**Systematic review registration:**

CRD42021220300.

## 1 Introduction

Stroke is the third most common cause of disability in the world and the number 1 killer in China.^[1]^ Some evidence indicated that stroke posed a serious threat to human health and life with high incidence and disability rates.^[2–5]^ Spasticity after stroke is a common dysfunction, causing pain, joint contractures and affecting the free movement of limbs.^[6–9]^ It is caused by damage to upper motor neurons and loss of control over spinal cord reflex activity. Nowdays, spasticity after stroke is a major problem for rehabilitation. According to recent studies, the incidence of spasticity ranged from 17% to 43% in poststroke patients.^[9–11]^

AHA/ASA adult stroke rehabilitation treatment guidelines recommended the treatment including oral drugs, normal limb position placement, and botulinum toxin injections.^[12]^ However, oral antispasmodic drugs can not selectively target the spasm site. The effect of botulinum toxin injection is short, which lasts no longer than 3 to 4 months. At the same time, it is not widely used the intrathecal injection of baclofen due to the small sample size of the study and the high technical requirements. Therefore, an increasing number of patients have sought alternative treatments.

Acupuncture is an important part of complementary and alternative medicine, which has a history of more than 3000 years in China. Acupuncture therapy is widely used in the treatment of spasticity after stroke due to confirmed efficacy and few adverse effects.^[13–15]^ Electroacupuncture, derived from the integration of traditional acupuncture and modern electrical stimulation, is another kind of acupuncture. After the needles are inserted into the acupuncture points, the electrodes are attached to the pairs of needles, and then a small electric current, usually with a pulse frequency of 1 to 100 Hz and pulse amplitude of 2 to 3 mA, is passed through the needles into the subject for 15 to 60 minutes.^[16]^ Compared with traditional manual acupuncture, electroacupuncture can provide a constant stimulation, whose intensity, frequency and duration are quantifiable.^[17,18]^ Therefore, electroacupuncture has become more and more widely used in clinical practice. Nowadays, there have been more and more studies on electroacupuncture in the treatment of spasticity after stroke. However, so far, as we all know, there is only 1 systematic review (SR) about electroacupuncture for patients with spasticity after stroke. Because the quality of reporting is low, and the author concluded that the clinical result was tentative because of a lack of high-quality evidence. Therefore, according to a rigorous review method, we intend to perform a SR to evaluate the effectiveness and safety of electroacupuncture for patients with spasticity after stroke. We hope that we could provide a convincing conclusion.

## 2 Methods and analysis

Our SR is designed in strict compliance with the preferred reporting items for systematic reviews and meta-analysis protocol (PRISMA-P).^[19]^ The PRISMA guidelines and the Cochrane Handbook will be used for us to evaluate the included studies. Besides, our SR will carry out bias risk analysis, heterogeneity analysis. If necessary, subgroup analysis and sensitivity analysis will be conducted. The protocol for this SR has been registered on PROSPERO with registration number: CRD42021220300.

### 2.1 Inclusion criteria

#### 2.1.1 Types of participants

Patients with diagnosed spasticity after stroke will be included, regardless of gender, age, race, education status, and cases of the source. All participants included in the SR must comply with the diagnostic criteria of stroke and symptoms of increased limb muscle tension.

#### 2.1.2 Types of interventions

Intervention measures should be electroacupuncture alone or combined with other methods to treat spasticity after stroke. If combined with other methods, only the control group with the same intervention measures as the experimental group will be included.

#### 2.1.3 Types of studies

Randomized controlled clinical trials and quasi-randomized controlled trials will be included. We will exclude any other types of literature including literature on electroacupuncture as nonmajor interventions, retrospective research literature, repeated publication literature, conference abstracts, literature that data cannot be extracted, case reports, and bibliometrics research. Owing to the language restriction of our researchers, we will limit the language of search literature to Chinese and English.

#### 2.1.4 Types of outcomes

The primary outcomes will be the modified Ashworth scale (MAS). The MAS will be used to evaluate muscle tone. The degree of spasm is divided into 0, I, I+, II, III, and IV levels according to the resistance felt by the evaluators during passive movement of the elbow and knee joints of hemiplegic patients in the resting state. The higher the score, the higher the degree of spasm.

The secondary outcomes will include the following measures:

1. Simplified Fugl–Meyer assessment scale (FMA);
2. Stroke specific quality of life scale (SS-QOL);
3. Adverse events

### 2.2 Data sources and search methods

#### 2.2.1 Electronic searches

This study will use computer search Medline, Embase, Pubmed, Web of science, and the Cochrane Central Register of Controlled Trials. In addition, we will also collect 4 databases of China: China National Knowledge Infrastructure, China Biomedical Literature Database, China Science Journal Database, and Wan-fang Database. All databases will be searched from the date of creation to November 2020. The following search terms will be used: spastic paralysis, spastic hemiplegia, spastic paraparesis, stroke, post-stroke, apoplex, acupuncture, meridian, electroacupuncture, electro acupuncture, electro-stimulation, etc. The example search strategy in Table 1 will be used for Pubmed. This search strategy will be slightly modified and used in several other databases.

**Table 1:** Search strategy used in PubMed.

No	Search items
#1	Spastic paralysis (all field)
#2	Spastic hemiplegia (all field)
#3	Spastic paraparesis (all field)
#4	Upper motor nearon paralysis (all field)
#5	Central paralysis (all field)
#6	Muscle spasticity (all field)
#7	Stiff paralysis (all field)
#8	Spastic (all field)
#9	Paralysis (all field)
#10	#1 OR #2-#9
#11	Stroke (all field)
#12	Post-stroke (all field)
#13	Apoplex (all field)
#14	Cerebrovascular disorder (all field)
#15	Brain ischemia (all field)
#16	Intracranial arterial disease (all field)
#17	Intracranial embolism and thrombosis (all field)
#18	Intracranial haemorrhages (all field)
#19	#11 OR #12-#18
#20	Acupuncture (all field)
#21	Acupoint (all field)
#22	Meridians (all field)
#23	Electroacupuncture (all field)
#24	Electro acupuncture (all field)
#25	Electro-stimulation (all field)
#26	#20 OR #21-#25
#27	Randomized controlled trial (all field)
#28	Controlled clinical trial (all field)
#29	Randomized (all field)
#30	Randomly (all field)
#31	#27 OR #28-#30
#32	#10 AND #19 AND #26 AND #31

#### 2.2.2 Searching other resources

We will search the list of the related references for additional trials. The PubMed and Cochrane Library will be searched for existing SRs related to our topic to search their reference lists for more studies. We will also search a reference list for identifying published journals, books, conference articles and gray literature related to this research topic.

### 2.3 Data collection and export

**Figure 1:**
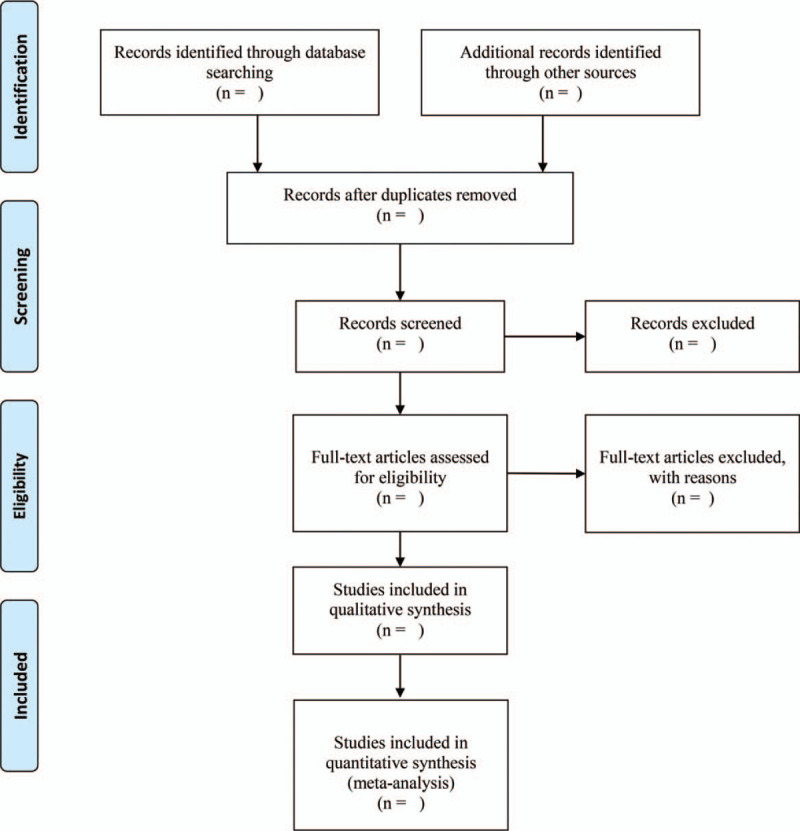
Quorum flow diagram for study retrieval and selection.

After completing all the search work, the results will be exported to Endnote software Version X9, and repetitive studies will be deleted by the software. The process of filtering documents will be completed independently by 2 reviewers and then cross-checked to determine the final inclusion of the documents. In the first stage, all the documents in the search results will be screened for titles, abstracts, and keywords to determine which tests meet the selection criteria. In the second stage, we will evaluate the full text of the study and determine whether it is eligible for SRs. Studies excluded after reading the full text will also be documented and explained why they were excluded. When differences arise at any stage, we will invite the third reviewer to discuss arbitration. The research flow chart is shown in Figure 1.

### 2.4 Data extraction and analysis

Data extraction and analysis will be done by 2 researchers independently, and the results will be cross-matched. When the differences and opinions are inconsistent, they should be settled through discussion. If the differences encountered cannot be resolved through discussion, a third author will be invited to resolve them. We will make an Excel to extract literature data, which includes the first author, country, year of publication, patient characteristics, number of participants, interventions, outcome, results, main conclusions, conflicts of interest, ethical approval, and other information. If the reported data are not sufficient, we will contact the author of the experiment for consultation and solution.

### 2.5 Assessment of risk of bias in the included studies

We will use the Risk of bias tool in Cochrane Manual V.5.1.0 to evaluate the bias risk of each included studies. The contents include: random sequence generation, allocation sequence concealment, blinding of participants and personnel and outcome assessors, incomplete outcome data, selective outcome reporting, and other sources of bias. The assessment results will be divided into 3 levels: low risk, high risk, and uncertain risk.

### 2.6 Assessment of heterogeneity

The heterogeneity of data will be tested by calculating the value of the *I*^2^ statistic. The study is not considered to have large heterogeneous when the *I*^2^ value is less than 50%. However, when the *I*^2^ value exceeds 50%, there is significant statistical heterogeneity among the trials, and meta-analysis will not be performed. At this time, subgroup stratification analysis is needed to explore the possible causes of heterogeneity.

### 2.7 Assessment of reporting biases

We will use funnel charts to assess reporting biases. When a sufficient number of included studies (at least 10 trials) are available, we will conduct a test for funnel plot asymmetry using the Egger method.

### 2.8 Data synthesis

The data synthesis will be performed by using the RevMan V.5.3. The results will be expressed as risk ratio and the standardized or weighted average difference of continuous data. The specific methods are as follows: If the *I*^2^ test is less than 50%, the fixed-effects model will be used for data synthesis. If the *I*^2^ test is between 50% and 75%, the random-effects model will be conducted for data synthesis. If the *I*^2^ test is higher than 75%, we will investigate possible reasons from both clinical and methodological perspectives to conduct subgroup analysis. If data cannot be synthesized, we will provide a descriptive analysis to solve this problem.

### 2.9 Subgroup analysis

In the case of high heterogeneity, we will conduct a subgroup analysis to identify the sources of heterogeneity. Besides, according to different course time, or other factors affecting the results, we will also make subgroup analysis.

### 2.10 Sensitivity analysis

In order to test the robustness of the main decisions in the review process, we will conduct a sensitivity analysis. The main analysis points include the impact of method quality, sample size, and missing data on the study. The meta-analysis will be reused, and more inferior quality studies will be excluded. The results will be compared and discussed according to the results.

### 2.11 Grading the quality of evidence

The quality of SRs will be evaluated by using the grading of recommendations assessment, development, and evaluation.^[20,21]^ Five downgrading factors including risk of bias, inconsistency, indirectness, imprecision, and publication bias will be assessed. The assessment results will be divided into 4 levels: high, moderate, low, or very low.

## 3 Discussion

As one of the most commonly seen complications of stroke.^[22]^ Spasticity can have a negative impact on around 20% to 50% of stroke survivors.^[7,23]^ The direct cost for patients with spasticity after stroke was reported nearly 4 times higher than those without it.^[24]^ The relatively short-term effects and heavy financial burden with current therapies were attached to great concern.^[25]^ Electroacupuncture, as a product of the combination of traditional acupuncture and modern medicine, enables clinicians to apply a standardized treatment in clinical practice and has no obvious side effects. And it is widely used in the treatment of spasticity after stroke.^[26,27]^ Electroacupuncture has been proved to protect central neurons by improving blood supply in ischemic areas of the brain and promoting the proliferation of central nerve cells, so as to achieve the functional restructuring of the central nervous system.^[27,28]^ However, due to lack structured methodological approach, there is still a need to establish valid evidence to support that electroacupuncture is effective for spasticity after stroke. Therefore, we will conduct an SR and meta-analysis to assess the effectiveness and safety of electroacupuncture for poststroke spasticity. It is hoped that the results of this SR may help to provide convincing evidence for patients and clinicians during the decision-making process.

## Author contributions

Kang Yang and Yufeng Wang contributed to the conception of the study. Kang Yang drafted and revised the manuscript. The search strategy was developed by all the authors and will be performed by Hongshi Zhang and Guanyu Hu, Ye Zhang and Huijuan Lou independently screen the potential studies, extract data from the included studies, assess the risk of bias and complete the data synthesis. Meng Meng will arbitrate in cases of disagreement and ensure the absence of errors. All authors approved the publication of the protocol.

**Data curation:** Ye Zhang.

**Formal analysis:** Huijuan Lou.

**Funding acquisition:** Deyu Cong.

**Investigation:** Hongshi Zhang, Meng Meng.

**Methodology:** Guanyu Hu.

**Validation:** Yufeng Wang.

**Writing – original draft:** Kang Yang.

**Writing – review & editing:** Yufeng Wang, Deyu Cong.

## References

[1] Zhou M Wang H Zeng X. Mortality, morbidity, and risk factors in China and its provinces, 1990-2017: a systematic analysis for the Global Burden of Disease Study 2017. Lanct 2019;394:1145–58. 10.1016/S0140-6736(19)30427-1 PMC6891889 31248666

[2] Morovatdar N Thrift Amanda G Stranges S. Socioeconomic status and long-term stroke mortality, recurrence and disability in Iran: the Mashhad stroke incidence study. Neuroepidemiology 2019;53:27–31. 30991387 10.1159/000494885

[3] GBD 2016 Neurology Collaborators. Global, regional, and national burden of neurological disorders, 1990-2016: a systematic analysis for the Global Burden of Disease Study 2016. Lancet Neurol 2019;18:459–80. 30879893 10.1016/S1474-4422(18)30499-X PMC6459001

[4] Reardon MJ Van Mieghem NM Popma JJ. Surgical or transcatheter aortic-valve replacement in intermediate-risk patients. N Engl J Med 2017;376:1321–31. 28304219 10.1056/NEJMoa1700456

[5] Feigin VL Roth GA Naghavi M. Global burden of stroke and risk factors in 188 countries, during 1990-2013: a systematic analysis for the Global Burden of Disease Study 2013. Lancet Neurol 2016;15:913–24. 27291521 10.1016/S1474-4422(16)30073-4

[6] Dietz V Sinkjaer T. Spastic movement disorder: impaired reflex function and altered muscle mechanics. Lancet Neurol 2007;6:725–33. 17638613 10.1016/S1474-4422(07)70193-X

[7] Zorowitz RD Gillard PJ Brainin M. Poststroke spasticity: sequelae and burden on stroke survivors and caregivers. Neurology 2013;80: (3 Suppl 2): S45–52. 10.1212/WNL.0b013e3182764c86 23319485

[8] Pundik S McCabe J Skelly M. Association of spasticity and motor dysfunction in chronic stroke. Ann Phys Rehabil Med 2019;62:397–402. 30099149 10.1016/j.rehab.2018.07.006

[9] Schinwelski MJ Sitek EJ Wąż P. Prevalence and predictors of post-stroke spasticity and its impact on daily living and quality of life. Neurol Neurochir Pol 2019;53:449–57. 31845749 10.5603/PJNNS.a2019.0067

[10] Mahmood A Veluswamy SK Hombali A. Effect of transcutaneous electrical nerve stimulation on spasticity in adults with stroke: a systematic review and meta-analysis. Arch Phys Med Rehabil 2019;100:751–68. 30452892 10.1016/j.apmr.2018.10.016

[11] Wissel J Manack A Brainin M. Toward an epidemiology of poststroke spasticity. Neurology 2013;80: (3 Suppl 2): S13–9. 10.1212/WNL.0b013e3182762448 23319481

[12] Winstein CJ Stein J Arena R. Guidelines for adult stroke rehabilitation and recovery: a guideline for healthcare professionals from the American Heart Association/American Stroke Association. Stroke 2016;47:e98–169. 27145936 10.1161/STR.0000000000000098

[13] Yang L Tan JY Ma H. Warm-needle moxibustion for spasticity after stroke: a systematic review of randomized controlled trials. Int J Nurs Stud 2018;82:129–38. 29631145 10.1016/j.ijnurstu.2018.03.013

[14] Wu D Qian L Zorowitz RD. Effects on decreasing upper-limb poststroke muscle tone using transcranial direct current stimulation: a randomized sham-controlled study. Arch Phys Med Rehabil 2013;94:1–8. 22878231 10.1016/j.apmr.2012.07.022

[15] Yue ZH Li L Chang XR. Zhongguo Zhen Jiu[Article in Chinese] 2012;32:582–6. 22997781

[16] Chen CY Ke MD Kuo CD. The influence of electro-acupuncture stimulation to female constipation patients. Am J Chin Med 2013;41:301–13. 23548121 10.1142/S0192415X13500225

[17] Zhou F Guo J Cheng J. Electroacupuncture increased cerebral blood flow and reduced ischemic brain injury: dependence on stimulation intensity and frequency. J Appl Physiol (1985) 2011;111:1877–87. 21836043 10.1152/japplphysiol.00313.2011 PMC3233896

[18] Wei JJ Yang WT Yin SB. The quality of reporting of randomized controlled trials of electroacupuncture for stroke. BMC Complement Altern Med 2016;16:512–1512. 27938353 10.1186/s12906-016-1497-y PMC5148866

[19] Moher D Shamseer L Clarke M. Preferred reporting items for systematic review and meta-analysis protocols (PRISMA-P) 2015 statement. Syst Rev 2015;4:1. 25554246 10.1186/2046-4053-4-1 PMC4320440

[20] Schünemann HJ Oxman AD Brozek J. Grading quality of evidence and strength of recommendations for diagnostic tests and strategies. BMJ 2008;336:1106–10. 18483053 10.1136/bmj.39500.677199.AE PMC2386626

[21] van de Griendt EJ Tuut MK de Groot H. Applicability of evidence from previous systematic reviews on immunotherapy in current practice of childhood asthma treatment: a GRADE (Grading of Recommendations Assessment, Development and Evaluation) systematic review. BMJ Open 2017;7:e016326. 10.1136/bmjopen-2017-016326 PMC5770836 29288175

[22] Miller EL Murray L Richards L. Comprehensive overview of nursing and interdisciplinary rehabilitation care of the stroke patient: a scientific statement from the American Heart Association. Stroke 2010;41:2402–48. 20813995 10.1161/STR.0b013e3181e7512b

[23] Kwah LK Harvey LA Diong JH. Half of the adults who present to hospital with stroke develop at least one contracture within six months: an observational study. J Physiother 2012;58:41–7. 22341381 10.1016/S1836-9553(12)70071-1

[24] Lundström E Smits A Borg J. Four-fold increase in direct costs of stroke survivors with spasticity compared with stroke survivors without spasticity: the first year after the event. Stroke 2010;41:319–24. 20044535 10.1161/STROKEAHA.109.558619

[25] Nair KP Marsden J. The management of spasticity in adults. BMJ 2014;349:g4737. 25096594 10.1136/bmj.g4737

[26] Mukherjee M McPeak LK Redford JB. The effect of electro-acupuncture on spasticity of the wrist joint in chronic stroke survivors. Arch Phys Med Rehabil 2007;88:159–66. 17270512 10.1016/j.apmr.2006.10.034

[27] Li J He J Du Y. Electroacupuncture improves cerebral blood flow and attenuates moderate ischemic injury via Angiotensin II its receptors-mediated mechanism in rats. BMC Complement Altern Med 2014;14:441. 25387826 10.1186/1472-6882-14-441 PMC4237754

[28] Hong J Wu G Zou Y. Electroacupuncture promotes neurological functional recovery via the retinoic acid signaling pathway in rats following cerebral ischemia-reperfusion injury. Int J Mol Med 2013;31:225–31. 23129018 10.3892/ijmm.2012.1166

